# Miscellaneous

**Published:** 1858-12

**Authors:** 


### Miscellaneous.

### REPORT OF THE EXECUTIVE COMMITTEE.

THE Executive Committee, appointed at the last "American Dental Convention," would respectfully report the following as the order of business for the next Convention which is to meet at "Niagara Falls," on the first Tuesday in August, 1859, at 10 o'clock, A. M.:

1. Reading and correcting report of the last meeting.
2. Treasurer's report.
3. Report of committees.
4. Election of officers.
5. The retiring President's address.
6. Installation of officers.
7. Appointing committees.

DISCUSSIONS.

1. When and under what circumstances should teeth be extracted, to secure a correct development of the permanent teeth?
2. Mechanical appliances and forces, to correct irregularities of the teeth.
3. Under what circumstances and in what manner should teeth be filed to remove superficial decay, or preparatory to filling, to leave them the most secure from future decay?
4. Filling teeth.
5. Mechanical dentistry.
6. Treament of diseases of the antrum.
7. Professional intercourse.

MISCELLANEOUS.

The reading of original essays on the various topics under discussion, will be first in order under their appropriate heads.

The committee would respectfully suggest to all who expect to be present, the benefit, as well as the *propriety*, of preparing notes (to be read before the convention, of their experience and observation on the various subjects named for discussion). Let these notes be carefully prepared, short, and to the point. In this way a large amount of valuable information may be collected for publication and thus be put on record for the benefit of the profession at large.

W. W. ALLPORT,
W. M. WRIGHT,
W. D. STONE,
FRANK FULLER,
A. M. LESLIE.

---

MISS. V. A. OF DENTAL SURGEONS.

Subjects for discussion, at the next annual meeting of the Mississippi Valley Association of Dental Surgeons:

1. Treatment of inflamed or sensitive dentine.
2. Mechanical dentistry.
3. Treatment of dental periostitis, and alveolar abcess.
4. To what extent are atmospheric plates applicable to partial sets of teeth.
5. Anæsthetic agents, use of, in the extraction of teeth.
6. Irregularities of teeth, how corrected.
7. Filling teeth.
8. Best means to secure a good denture.

J. P. ULREY,
H. A. SMITH,
C. N. WOODWARD.

---

MISCELLANEOUS.

We have received the above list of subjects, from the business committee, for consideration at the next meeting of the Mississippi Valley Association of Dental Surgeons.

The subjects are all important, and should receive attention, by every practicing dentist. It may be said, that the most of these subjects have been discussed before; this is true; but then, all admitted that there is much yet to be learned about each of them, and every one felt regret that we know so little about them. And now, we have to keep working away at these things, till we do understand them fully; for these are the important points in dental practice.

ASSOCIATIONS.

The Mississippi Valley Association of Dental Surgeons meets according to adjournment, on Wednesday, Feb. 23d, 1859, at 10 o'clock, A. M., in the lecture room of the Ohio College of Dental Surgery,—at which time and place, we hope to see all the members of the profession, who can possibly make it convenient to attend. These meetings are always fraught with interest and profit to those who engage actively in them. We hope to see all the members of the society present, and also to have an addition of many, who, as yet, have not been identified with it.

The Ohio Dental College Association meets on the third Monday of February, 1859, at 2 o'clock, P. M., at the lecture room of the College.

It is very desirable that all the members of the Association be present. All the stockholders should feel interested enough, to be present at its annual meetings, and assist in the transaction of its business.

The new Certificates are ready for delivery to the stockholders, and those holding old Certificates will please bring them in, that they may be exchanged for the new. ☞ It is very desirable to have them all in, at the next meeting.

The Commencement exercises of the Ohio College of Dental Surgery, will take place at the College, on the evening of Wednesday, Feb. 23d, 1859, at 7½ o'clock.

☞ The 4th Annual meeting of the Michigan Dental Association will be held at the Office of Drs. Whiting & Benedict, in the City of Detroit, on Tuesday, the 11th day of January next, at 7 o'clock P. M.

As many important changes and improvements have been made in the Profession during the past year, a full attendance of the members of the Association is expected.

The advancement of Dental Science, and the general interests of the Profession being the principle objects of the Association, it is hoped that every regular practicing Dentist in the State, will also be present at the next meeting. Respectfully yours, A. T. METCALF, Sec'y.

